# Optical Properties of Colloidal Silver Nanowires

**DOI:** 10.1021/acs.jpcc.2c01251

**Published:** 2022-05-17

**Authors:** Ruben F. Hamans, Matteo Parente, Aitzol Garcia-Etxarri, Andrea Baldi

**Affiliations:** † Department of Physics and Astronomy, Vrije Universiteit Amsterdam, De Boelelaan 1081, 1081 HV Amsterdam, The Netherlands; ‡ Dutch Institute for Fundamental Energy Research (DIFFER), De Zaale 20, 5612 AJ Eindhoven, The Netherlands; ¶ Donostia International Physics Center (DIPC), Manuel Lardizabal Ibilbidea 4, 20018 Donostia, Euskadi, Spain; § IKERBASQUE, Basque Foundation for Science, 48013 Bilbao, Euskadi, Spain

## Abstract

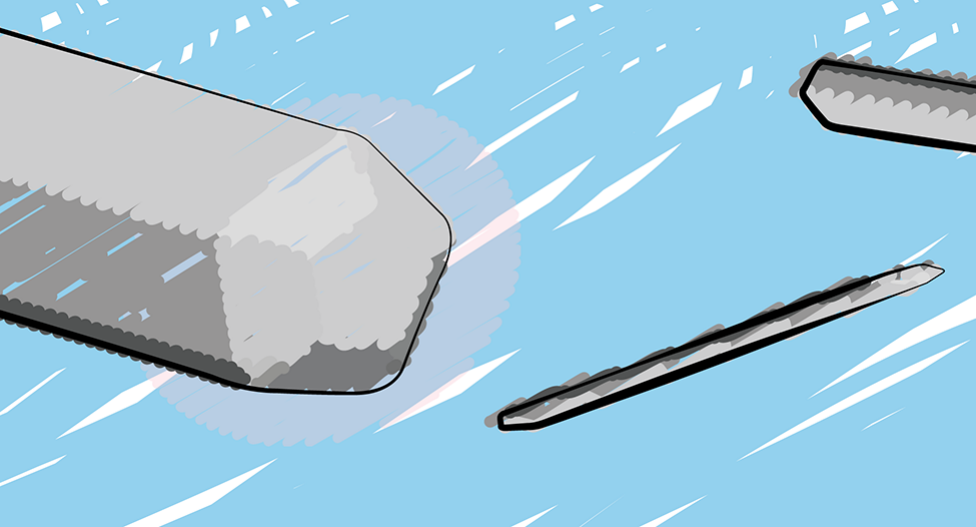

Silver nanowires are used in many applications, ranging from transparent conductive layers to Raman substrates and sensors. Their performance often relies on their unique optical properties that emerge from localized surface plasmon resonances in the ultraviolet. To tailor the nanowire geometry for a specific application, a correct understanding of the relationship between the wire’s structure and its optical properties is therefore necessary. However, while the colloidal synthesis of silver nanowires typically leads to structures with pentagonally twinned geometries, their optical properties are often modeled assuming a cylindrical cross-section. Here we highlight the strengths and limitations of such an approximation by numerically calculating the optical and electrical response of pentagonally twinned silver nanowires and nanowire networks. We find that our accurate modeling is crucial to deduce structural information from experimentally measured extinction spectra of colloidally synthesized nanowire suspensions and to predict the performance of nanowire-based near-field sensors. On the contrary, the cylindrical approximation is fully capable of capturing the optical and electrical performance of nanowire networks used as transparent electrodes. Our results can help assess the quality of nanowire syntheses and guide in the design of optimized silver nanowire-based devices.

## Introduction

Silver nanowires (AgNWs) and nanowire networks show high optical transparency in the visible range together with high electrical conductivity, making them appealing for a variety of applications, ranging from transparent electrodes^1−3^ to pressure, temperature, and strain sensors,^4−6^ substrates for Raman spectroscopy,^7^ and catalysis.^8,9^ As their optical and electrical properties strongly depend on their size and shape,^10−12^ it is of paramount importance to tailor their dimensions to the intended application. Numerical simulations allow prediction of the optical response of AgNWs and can therefore guide the design of nanowire-based optoelectronic devices. Usually, the optical extinction cross-sections of silver nanowires are simulated by approximating them to ellipsoids or to infinitely long cylinders using Mie theory.^13−17^ However, the typical method by which AgNWs are produced, the so-called polyol synthesis,^18,19^ leads to nanowires with pentagonal cross-sections and rounded edges.^2,20−22^ This difference between simulated and synthesized geometries leads to the prediction of extinction spectra that are inaccurate and miss crucial optical features. Moreover, the distribution and intensity of the scattered electric fields surrounding the nanowires, the so-called near-fields, are strongly shape dependent.^10^ The use of proper geometrical models of the nanowires is therefore important for all applications relying on an accurate prediction of the near-fields, such as surface enhanced Raman scattering (SERS), photocatalysis, and optical sensing.^23−27^

Here we use a finite difference time domain (FDTD) method to calculate the light scattering, absorption, and extinction of AgNWs with realistic pentagonal cross-sections. The simulated extinction spectra accurately reproduce all key features observed experimentally for colloidally synthesized nanowires. We show that the residual extinction in the visible range is a physical limit due to the geometry of the system and not, as often assumed, an indication of the presence of synthetic byproducts. Interestingly, the number and relative intensity of the plasmonic peaks in the ultraviolet range are extremely sensitive markers of the nanowire diameter and of the radius of curvature of their edges. On the contrary, we show that silver nanowire networks used as transparent electrodes have optical transparencies and electrical conductivities that are insensitive to the exact shape of the modeled nanowires and mainly depend on the magnitude of their geometrical cross-section. Finally, we compare near-field maps for circular and pentagonal cross-sections and highlight the importance of a proper model of the nanowire shape to predict field enhancements.

## Methods

### Mie Theory

For the case of infinitely long cylinders, analytical solutions to Maxwell’s equations exist in the form of Mie theory, which allows us to decompose the extinction spectrum into dipolar, quadrupolar, and higher order contributions. Mie theory calculations are performed using MatScat^28^ with a dielectric function from the literature.^29^

### FDTD Simulations

**Figure 1:**
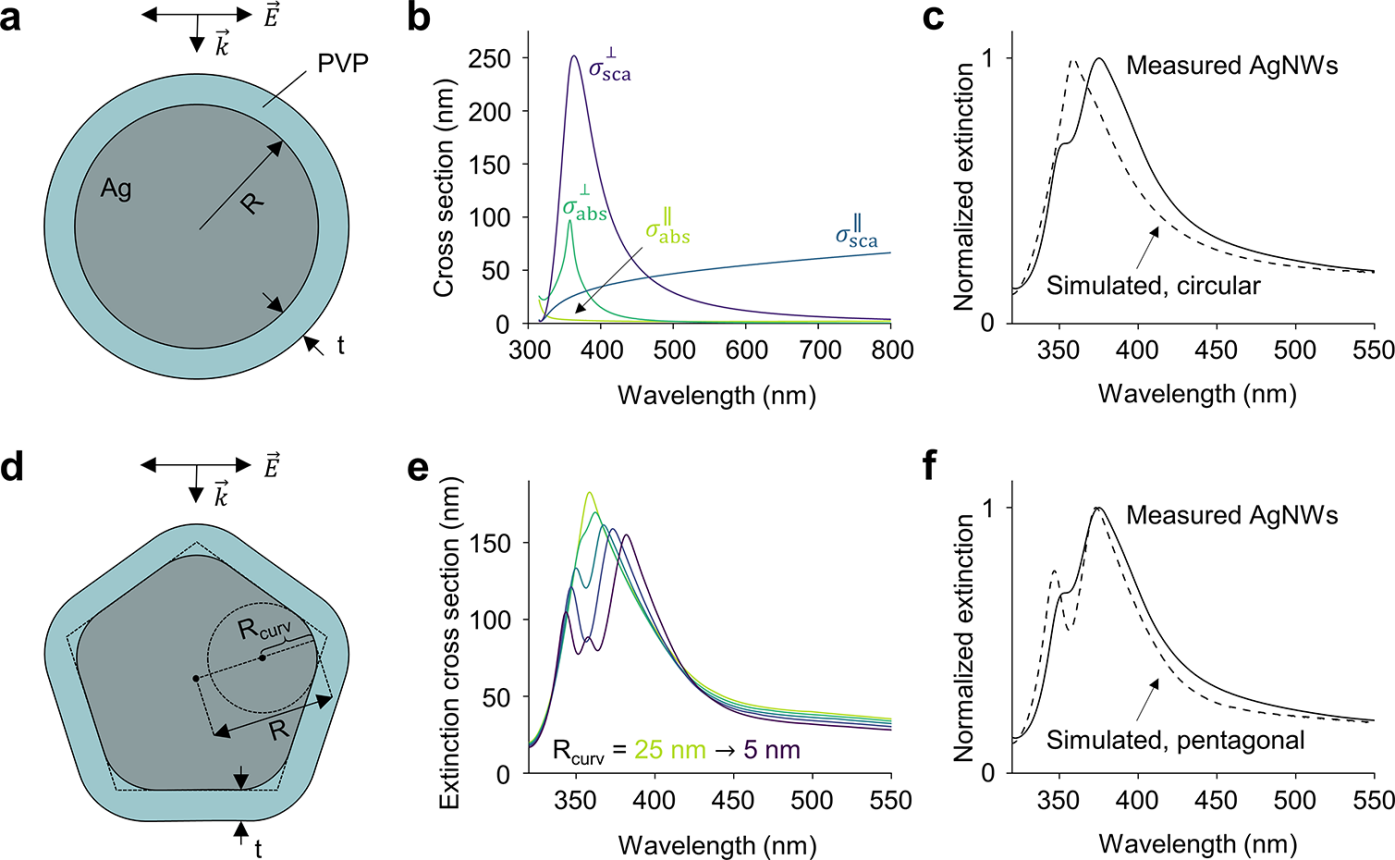
Optical response of infinitely long silver nanowires with circular and pentagonal cross-sections. (a) Illustration of a circular infinite nanowire of radius *R* surrounded by a PVP layer of thickness *t* under perpendicular illumination. (b) Simulated scattering and absorption cross-sections under parallel and perpendicular illumination for a circular infinite nanowire with *R* = 25 nm and *t* = 5 nm. (c) Comparison between the simulated (dashed) extinction spectrum of a circular infinite nanowire and the one measured experimentally for 25 nm radius PVP-stabilized AgNWs in water (solid).^31^ The simulated extinction cross-section is obtained by averaging over the two incoming parallel and perpendicular polarizations shown in panel b. (d) Illustration of a pentagonal infinite nanowire of radius *R* and radius of curvature *R*_curv_. (e) Simulated extinction cross-sections of pentagonal infinite nanowires with *R* = 25 nm and *R*_curv_ varying from 25 nm (green) to 5 nm (purple) in steps of 5 nm. The cross-sections are averaged over the two incoming polarizations *E⃗*_∥_ and *E⃗*_⊥_. (f) Comparison between the simulated (dashed) extinction spectrum of a pentagonal infinite nanowire (*R*_curv_ = 10 nm) with the experimental one shown also in panel c (solid).

FDTD simulations are performed using Lumerical FDTD^30^ with a dielectric function from the literature.^29^ The nanowire has a pentagonal cross-section (see main text and Figure 1d) and is assumed to be infinitely long due to the use of a two-dimensional simulation geometry. The simulation bandwidth ranges from 315 to 800 nm to ensure an accurate fit of the dielectric function over all simulated wavelengths (Figure S1). Around the AgNW a fine mesh of 0.25 × 0.25 nm^2^ is used.

### Sheet Resistance Model

The sheet resistance is modeled using a previously reported model.^16^ The input variables (wire diameter, wire length, wire density, and simulation box size) are mentioned in the main text. The model maps the spatial coordinates of the nanowire junctions and assigns either a junction with a resistance *R*_junc_ or a segment with a resistance *R*_seg_. The corresponding resistance matrix is solved using Kirchhoff’s circuit law. To obtain accurate sheet resistances and their corresponding standard deviations, the number of samples is varied from 3, for high nanowire densities, to 100, for low nanowire densities.

## Results and Discussion

### Comparison between Measured and Simulated Extinction Spectra

We first compare the experimental extinction spectrum of a solution of 25 nm radius AgNWs stabilized by polyvinylpyrrolidone (PVP) and suspended in water^31^ to the one calculated with two-dimensional Mie theory assuming an infinitely long cylinder surrounded by a homogeneous medium (Figure 1a).^28,32^ The silver dielectric function is taken from the literature,^29^ and the refractive indices of PVP and water are 1.56 and 1.333, respectively. The thickness of the PVP surfactant layer is 5 nm.^33^ The polarization of the incoming field is either parallel (*E⃗*_∥_) or perpendicular (*E⃗*_⊥_) to the wire.

For a parallel illumination, we observe a near-zero absorption cross-section σ_abs_ in the visible region that rises for λ < 350 nm thanks to interband transitions in silver (Figure 1b).^34^ The scattering cross-section σ_sca_ shows a broadband response for λ > 350 nm, similar to the reflectivity of an Ag mirror (Figure S2). For a perpendicular illumination, both σ_abs_ and σ_sca_ show peaks in the ultraviolet (UV) region corresponding to the transverse plasmon resonance of the wire (Figure 1b).

From σ_abs_ and σ_sca_ we define the extinction cross-section σ_ext_ = σ_abs_ + σ_sca_. In colloidal suspensions, the AgNWs are randomly oriented with respect to the polarization of the incoming light. To reproduce the measured optical properties of colloidal silver nanowires, we therefore average σ_ext_ over the two incoming polarizations *E⃗*_∥_ and *E⃗*_⊥_. The resulting extinction spectrum is shown in Figure 1c and shows two notable features. First, the wire shows an extinction peak in the UV region corresponding to the transverse plasmon resonance. This resonance also appears in the experimental extinction spectrum (solid line in Figure 1c). However, the cylindrical shape is not able to accurately reproduce the characteristic double peak in the UV region that is typically observed experimentally.^31,35−39^ Varying the radius of the infinite cylindrical wire merely results in a shift of the transverse plasmon resonance peak but not in the appearance of a double peak (Figure S3).

Second, the wire exhibits significant extinction for wavelengths λ > 500 nm (Figures 1c and S4), which severely limits the transparency that can be achieved when using these wires as transparent electrodes. This residual optical extinction in the visible region is often attributed to the presence of byproducts in the colloidal synthesis of AgNWs.^20,36,40^ While byproducts such as spherical particles can certainly contribute to visible light scattering and absorption, here we show that light extinction also emerges as a physical limit of the nanowire system, caused by the Ag mirror-like scattering when the incoming polarization is parallel to the wire. The limited visible transparency can, therefore, not be indefinitely improved by purifying the products at the end of the synthesis.

To more accurately reproduce the pentagonal geometry of the AgNWs, we perform simulations using a finite-difference time-domain method (see Methods). We use the same dielectric function,^29^ which is now fit to a set of functions that satisfy the Kramers–Kronig relations (Figure S1). Figure 1d shows the typical pentagonally twinned cross-section of an AgNW with radius *R*. We also introduce the radius of curvature *R*_curv_ to account for smooth nanowire edges. To observe the influence of the pentagonal geometry, we vary *R*_curv_ from *R*_curv_ = *R* = 25 nm (perfect cylinder) to *R*_curv_ = 5 nm (pentagon with sharp corners) in steps of 5 nm, while maintaining *R* = 25 nm. As can be seen in Figure 1e, the decrease in *R*_curv_ first results in a redshift of the resonance and the appearance of a lower wavelength shoulder (*R*_curv_ = 20 nm). The resonance then further redshifts, and the lower wavelength shoulder becomes a well-defined peak (*R*_curv_ = 15–10 nm), until eventually the spectrum splits even further into three peaks (*R*_curv_ = 5 nm). The comparison with a typical experimental extinction spectrum clearly indicates that polyol-made AgNWs have pentagonal cross-sections with partially smoothed edges (Figures 1f and S3). The agreement between measured and simulated spectra demonstrates how UV–vis spectroscopy, when coupled to proper optical modeling, can be a powerful tool in assessing the quality of AgNW syntheses. For example, the quantity of synthetic byproducts can be properly estimated by comparing the UV extinction at the transverse resonance of the wires with the one measured in the visible. Furthermore, the radius of curvature of the wires’ edges, which is a crucial parameter for near-field applications, can be determined with almost nanometer precision by looking at the shape and spectral position of the transverse resonance peaks. Such an accurate structural characterization would otherwise only be possible with the most advanced electron microscopy techniques.

### Nanowire Networks as Transparent Electrodes

**Figure 2:**
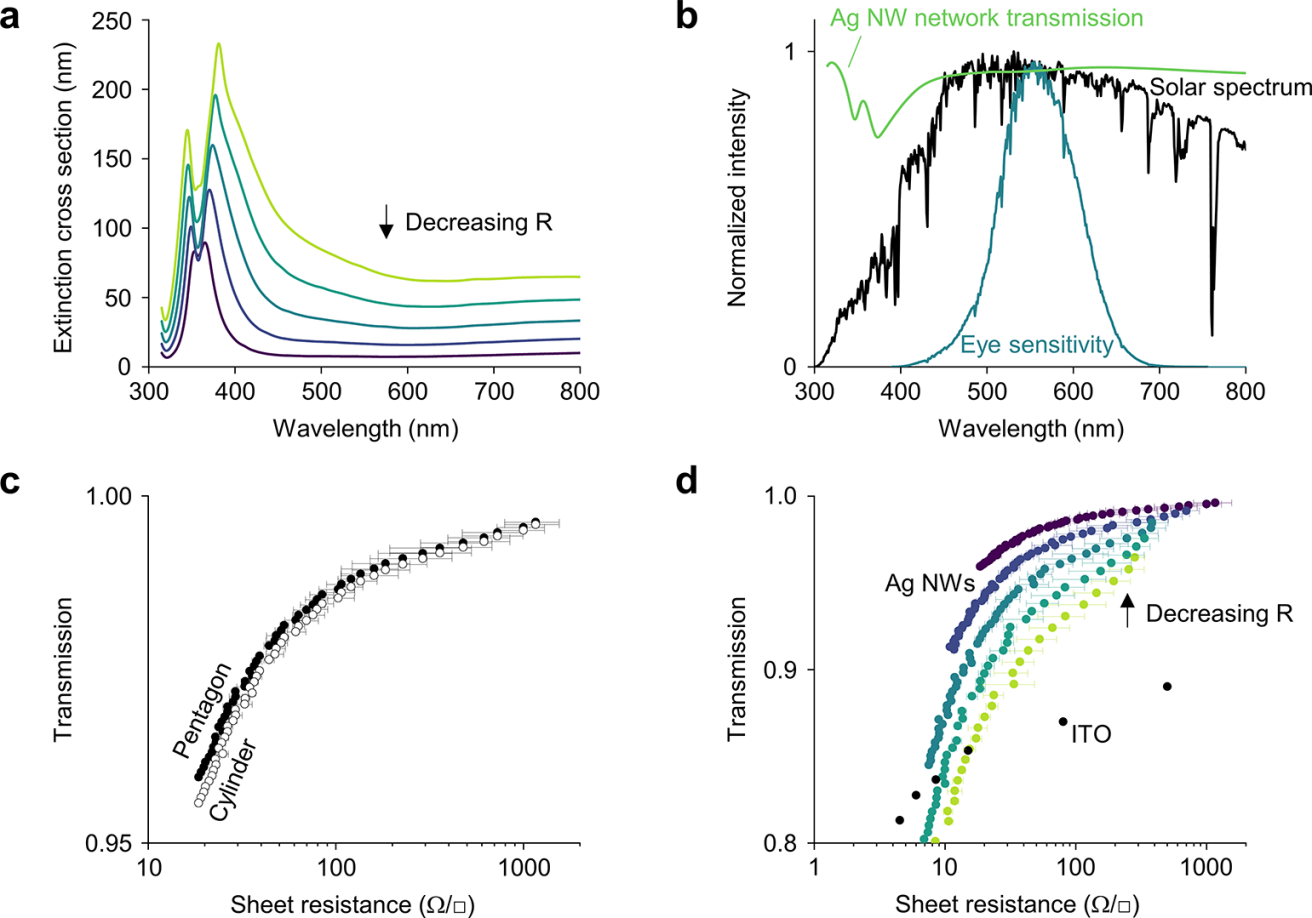
AgNWs for transparent electrodes. (a) Calculated extinction cross-section of an infinite pentagonal wire. The radius *R* is decreased from 35 to 15 nm in steps of 5 nm. The radius of curvature *R*_curv_ = 10 nm. (b) Solar spectrum before (black) and after (blue) multiplying by the human eye sensitivity, and an example of the transmission *T*_network_(λ) of a AgNW network. (c) Transmission and sheet resistance for AgNW networks consisting of wires with a pentagonal (closed circles) or circular (open circles) cross-section. (d) Transmission and sheet resistance of networks of pentagonal AgNWs with varying radii, as indicated using the same color scheme as panel (a). In panels c and d, every point is a different wire density, which ranges from 0.05 μm^–2^ to 0.55 μm^–2^. The junction resistance is 11 Ω, and the wire length is 10 μm. The error bars are standard deviations resulting from simulating multiple samples (see Methods).

Our improved optical model allows us to give several design rules for the use of AgNWs in specific applications. In particular, due to their plasmon resonance outside the visible region and their high electrical conductivity, networks of AgNWs can be used as transparent electrodes for smart windows, touch screens, solar cells, and organic light-emitting diodes (OLEDs).^41^ For these applications, a minimal extinction in the visible region is desired, while retaining a high conductivity of the network. Upon decreasing the radius of the wire, we observe a blueshift of the transverse plasmon resonance, together with a narrowing of the peak (Figure 2a). This blueshift toward the UV region of the spectrum has been used as a justification for the need of synthesizing thinner nanowires for applications in transparent electrodes.^42^ Although this strategy is correct, it can be seen from Figure 2a that the blueshift of the extinction is only a few nanometers. The largest transparency gain upon decreasing the nanowire radius is due to the lower residual extinction above 500 nm which results from the decreased geometrical size of the wire.

To quantify the performance of AgNW networks in the context of transparent electrodes, we calculate the optical transparency and electrical sheet resistance as a function of the wire density and wire diameter. To calculate the transparency, we first pick an appropriate source spectrum *I*_0_(λ). If we take the example of smart windows, where the electrode needs to be transparent to the human eye, we can define *I*_0_(λ) as the product between the solar spectrum and the response of a typical human eye to light (Figure 2b). We then calculate the wavelength-dependent optical transmission of the network *T*_network_(λ) using^17^
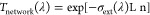
1where σ_ext_ is the two-dimensional extinction cross-section in meters, *L* is the length of the wire in meters, and *n* is the wire density in the number of wires per square meter. The integrated transmission *T* of the network is then expressed by
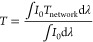
2Note that due to the distinct spectral shape of the human eye response (Figure 2b), we expect *T* to be similar for all applications where the human eye is the sensor, such as touch screens and OLEDs. For solar cell applications, however, the electrode needs to be transparent to photon energies above the bandgap of the semiconductor. In this latter case, a more appropriate choice for *I*_0_(λ) would be the portion of the solar spectrum with photon energies above the bandgap. For the most widely used semiconductor Si with a bandgap of 1.12 eV, this portion also includes the ultraviolet, which overlaps with the extinction peaks of the AgNW network. Therefore, for Si solar cells, we obtain slightly lower transmission values than for smart windows, touch screens, and OLEDs (Figure S5).

We also calculate the electrical sheet resistance.^16^ The sheet resistance of an AgNW network has contributions from the resistance at the junctions between different NWs and from the resistance of the NW segments between the junctions. When the junctions have poor electrical conductivity, the sheet resistance of the network is merely determined by the number of junctions.^13^ For the same wire length, a smaller radius results in an increased optical transparency (Figure 2a) but does not influence the amount of junctions and, therefore, also does not influence the sheet resistance, making the design recommendation straightforward. In practical applications, however, AgNW networks are often treated after deposition using, for example, thermal treatment^43^ or mechanical pressing^44^ to minimize junction resistance to the point where the internal resistance of the wires can no longer be neglected.^16,45,46^ As the resistance of a single wire scales with the inverse of its cross-sectional area, a small radius is preferred for highly transparent networks and a larger radius for highly conductive networks.

Using a previously reported model that has been validated with experimental data, we obtain the sheet resistance in Ohm per square, here denoted using Ω/□ (see also Methods).^16^ We model a 30 × 30 μm^2^ area with contacts on either side along the whole edge. Wires with a length *L* and a cross-sectional area *A* are placed randomly at a density *n*. Each junction has a resistance *R*_junc_, and the segments between the junctions have a resistance *R*_seg_ that is calculated using
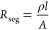
3where ρ = 2.26 × 10^–8^ Ωm is the resistivity of silver^47^ and *l* is the length of the segment. We find that for fairly poor junctions (*R*_junc_ = 1 kΩ), the sheet resistance is indeed junction-dominated and depends mostly on the wire density *n* and only weakly on the wire radius *R* (Figure S6). For optimized junctions (*R*_junc_ = 11 Ω), however, we find that an increased radius significantly decreases the total sheet resistance of the network (Figure S6).

For these optimized junctions, we first compare infinite cylindrical and pentagonal wires by modeling the sheet resistance and transmission of networks consisting of wires with equal cross-sectional areas (Figure S7). For example, we compare a pentagonal wire with *R* = 15 nm and *R*_curv_ = 10 nm to a cylindrical wire with *R* = 14.6 nm. The characteristic extinction peaks that differentiate these shapes lie in the UV part of the spectrum (Figure 1) where solar irradiation is not intense and where the human eye is not sensitive (Figure 2b). Therefore, when calculating *T*, the resulting transmission values are similar for pentagonal or circular geometries (Figure 2c). Due to this similarity, when altering the NW radius, our improved optical model yields results that agree with what has been reported previously for cylindrical wires (Figure 2d).^16^ For touch screen applications, a sheet resistance below ∼100 Ω/□ is sufficient. In these cases, AgNW networks outperform indium tin oxide (ITO) already at moderate NW densities, especially for small radii (Figure 2d). Furthermore, to obtain these sheet resistances, the junction resistance does not need to be optimized down to 11 Ω but is allowed to be higher (Figure S6). For OLEDs and solar cell applications, which require a lower sheet resistance of ∼10 Ω/□, the use of optimized junctions (Figure S6) and thin wires (Figure 2d) are instrumental.

### Nanowires as Near-Field Platforms

Whereas the transmission of a AgNW network mostly depends on the geometrical size of the AgNW rather than its exact shape, the spatial distribution and intensity of the electric field around the wire is expected to be strongly dependent on the nanowire cross-section and on the radius of curvature at its edges. Therefore, when estimating the performance of AgNWs for applications where the electric field strength is a key figure of merit, such as in Raman spectroscopy, catalysis, and sensing,^23−27^ it is important to simulate the right geometry and to understand which optical modes are supported by the AgNW and how these contribute to the near-field intensity and distribution.

**Figure 3:**
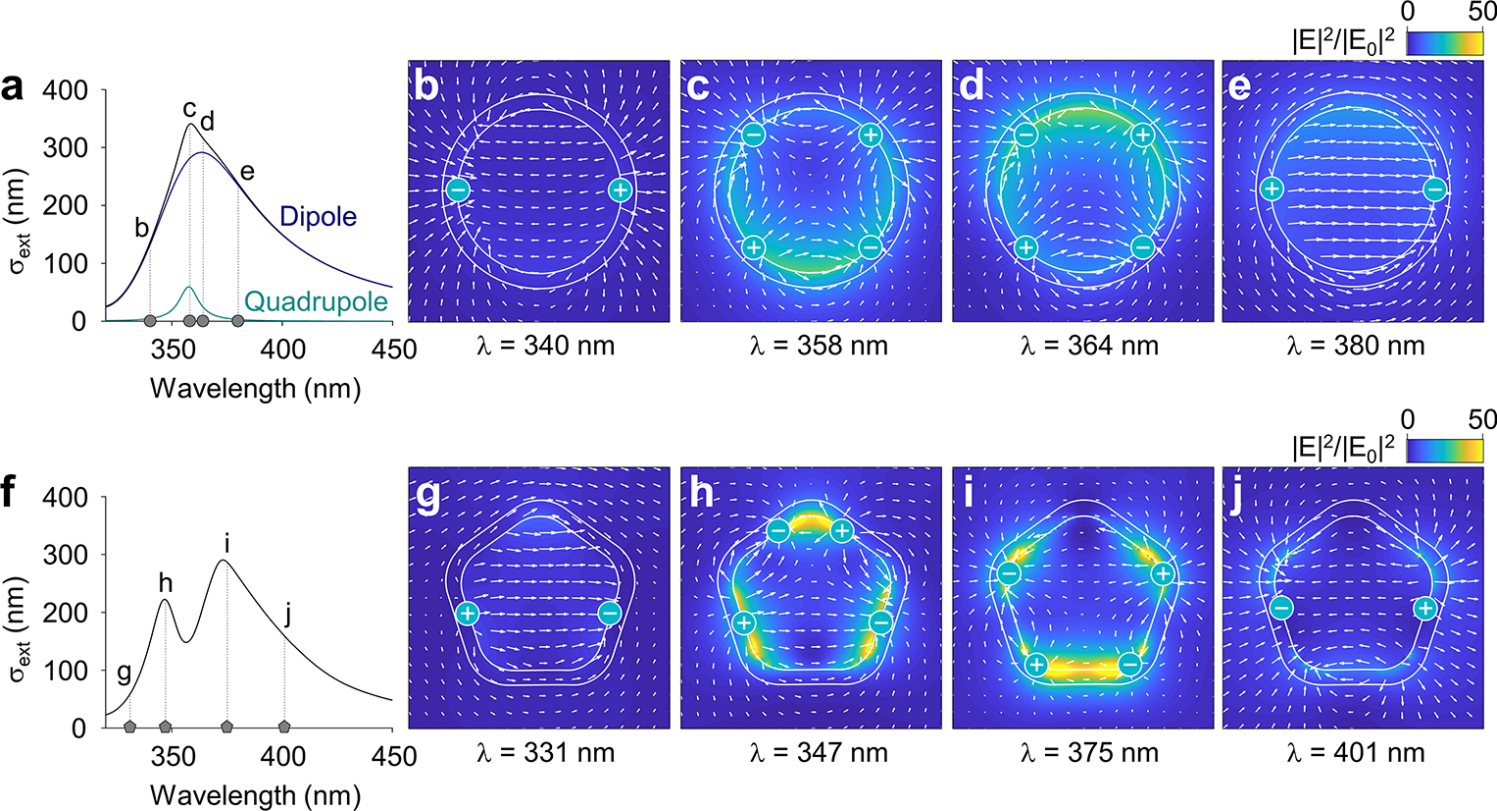
Extinction cross-sections and electric field distributions for infinite cylindrical and pentagonal wires. (a) Extinction cross-section calculated for an infinite cylindrical silver wire with a radius of 25 nm (black) and its decomposition into dipolar (dark blue) and quadrupolar (light blue) contributions. The dashed vertical lines correspond to the wavelengths of the field enhancement maps in the following panels: (b) 340 nm, (c) 358 nm, (d) 364 nm, and (e) 380 nm. (f) Extinction cross-section calculated for an infinite pentagonal silver wire with a radius of 25 nm and a radius of curvature of 10 nm. The dashed vertical lines correspond to the wavelengths of the field enhancement maps in the following panels: (g) 331 nm, (h) 347 nm, (i) 375 nm, and (j) 401 nm. The white arrows in the field enhancement maps indicate the real components of the vectorial electric field, and the plotted fields are total fields (incident + scattered).

The characteristic UV extinction peaks of AgNWs are associated with the excitation of transverse plasmon resonances (Figure 1a,b). In Figure 3, we compare the electric field distributions for infinite cylindrical and pentagonal wires under perpendicular light polarization. For infinite cylindrical wires, we use two-dimensional Mie theory,^28,32^ while for infinite pentagonal wires, we obtain the electric fields via FDTD simulations. In the former case, the contributions to the electric field and to the extinction spectrum can be decomposed into dipolar, quadrupolar, and higher order modes. Such decomposition cannot be performed using the FDTD method. We find that for *R* = 25 nm, besides the dipolar mode, the quadrupolar mode also contributes to the extinction spectrum (Figure 3a). In fact, the energy range of the transverse resonances observed here is also the range in which higher order modes in large Ag nanoparticles occur.^48^ However, contrary to nanoparticles, here the dipolar and quadrupolar modes appear at similar wavelengths and, therefore, do not appear as distinct peaks in the extinction spectrum. For wavelengths below the extinction maximum (λ < 358 nm), the quadrupole contribution is negligible (Figure 3a), resulting in near-field enhancement distributions with a dominant dipolar character, as indicated by the two opposite charges at the nanowire surface (Figure 3b). At the cylindrical wire resonance, both the quadrupolar (λ = 358 nm) and the dipolar (λ = 364 nm) resonances contribute to the overall extinction, resulting in an overall near-field distribution with a quadrupolar character (Figure 3c,d). For wavelengths above the extinction maximum (λ > 364 nm), the quadrupole contribution vanishes and the near-field enhancement distribution has again a simple dipolar character (Figure 3e).

The finite-difference time-domain method does not allow us to decompose the extinction spectrum into dipolar and quadrupolar contributions easily. However, in the near-field maps we observe two nondegenerate quadrupolar modes for both extinction peaks of the pentagonally twinned wire, as shown in Figure 3h,i, even though the low energy peak around 375 nm is often attributed to a dipolar resonance.^20,49^ This confusion likely stems from the analogy with the optical properties of large metallic spheres, in which the strong dipolar peak is accompanied by a smaller quadrupolar peak at a lower wavelength. For wavelengths above and below the extinction maxima, we again observe a dipolar near-field distribution (Figure 3g,j). Most notably, in the transition from a cylinder to a pentagonally twinned wire we also observe roughly a 2-fold increase in the electric field strength, which can be attributed to the presence of sharper corners (Figure 3).^50^ This observation highlights the importance of using an accurate geometrical description when predicting the performance of AgNW-based devices that rely on near-field enhancements.

## Conclusion

In summary, we studied how the optical properties of AgNWs depend on the shape of their cross-section. We demonstrated that comprehensive knowledge of the relationship between the optical properties and the geometry of AgNWs allows us to extract accurate structural information from simple UV–vis spectroscopy. We showed that the characteristic double extinction peak of colloidal AgNWs is a clear marker of a pentagonal cross-section and that the exact shape of the peak is extremely structurally sensitive to the radius of curvature of the edges, which is a crucial structural parameter for accurately modeling near-fields. On the contrary, when modeling AgNWs for transparent electrode applications, a simple cylindrical approximation is sufficient to reproduce the optical and electrical performance of nanowire networks. Our results can help in assessing the yield of AgNW syntheses, as well as in choosing the right nanowire dimensions for maximizing the sensing and enhancing effects in applications such as Raman spectroscopy.

## Supporting Information Available

Fit of the dielectric function; reflectivity of a Ag mirror, extinction of Ag cylinders with varying radii, full bandwidth comparison between circular and pentagonal cross-sections, AgNW networks for solar cells, AgNW network sheet resistance as a function of wire density, and pentagonal vs circular cross-section comparison (PDF)
