# Supplementary material for: Optical Properties of Colloidal Silver Nanowires
Source: J Phys Chem C Nanomater Interfaces. 2022 May 17;126(20):8703–9. doi: 10.1021/acs.jpcc.2c01251 (PMC9150108; doi:10.1021/acs.jpcc.2c01251)
Supplement: Supplementary file 1 — jp2c01251_si_001.pdf [file jp2c01251_si_001.pdf]

Supporting Information
On the Optical Properties of Colloidal Silver Nanowires

Ruben F. Hamans,[†,‡,‖] Matteo Parente,[‡,‖] Aitzol Garcia-Etxarri,[¶,§] and Andrea Baldi[*,†,‡]

†*Department of Physics and Astronomy, Vrije Universiteit Amsterdam, De Boelelaan 1081, 1081 HV Amsterdam, The Netherlands*

‡*Dutch Institute for Fundamental Energy Research (DIFFER), De Zaale 20, 5612 AJ Eindhoven, The Netherlands*

¶*Donostia International Physics Center (DIPC), Manuel Lardizabal Ibilbidea 4, 20018 Donostia, Euskadi, Spain*

§*IKERBASQUE, Basque Foundation for Science, 48013 Bilbao, Euskadi, Spain*

‖*Contributed equally to this work*

E-mail: a.baldi@vu.nl

Contents

1 Fit of the dielectric function **S3**

2 Reflectivity of an Ag mirror **S4**

3 Extinction of Ag cylinders with varying radii **S5**

4 Full bandwidth comparison between circular and pentagonal cross sections **S6**

5 AgNW networks for solar cells **S7**

6 AgNW network sheet resistance as a function of wire density **S8**

7 Pentagonal vs circular cross section comparison **S9**

References **S11**

1 Fit of the dielectric function

For the finite-difference time-domain (FDTD) simulations the experimental dielectric function is fitted (Figure S1). As FDTD simulations take place in the time domain, the relation between the dielectric displacement and the electric field needs to be causal, *i.e.* materials cannot respond to fields in the future. This condition places some restrictions on the fitted dielectric function, known as the Kramers-Kronig relations.[1]

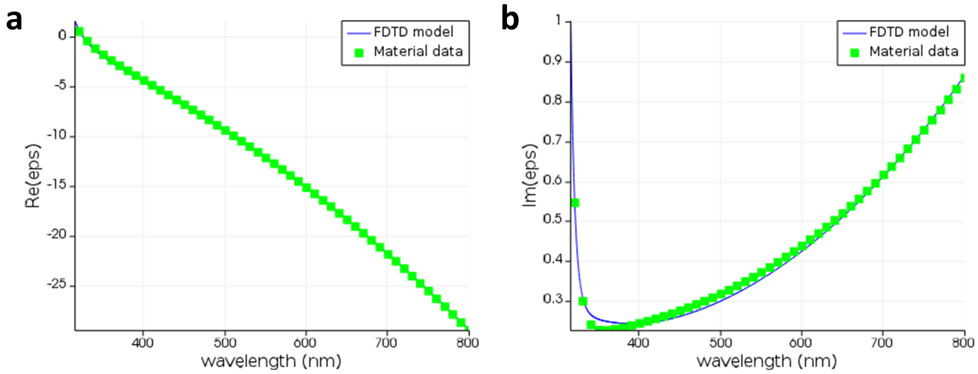

Figure S1: Fitted dielectric function for FDTD simulations. (a,b) Real (a) and imaginary (b) part of the experimental dielectric function (green squares) and the fit for the FDTD simulations (blue lines).

2 Reflectivity of an Ag mirror

For polarizations parallel to the long axis of the wire ($\vec{E}_{\parallel}$) we observe a broadband response in the scattering cross section σ_{sca} of an Ag cylinder (Figure 1 of the main text). To demonstrate the analogy with the reflectivity of an Ag mirror we simulate the transmission, reflection, and absorption of a 50 nm Ag film (Figure S2). We use a finite-difference time-domain method with a dielectric function taken from literature.[2]

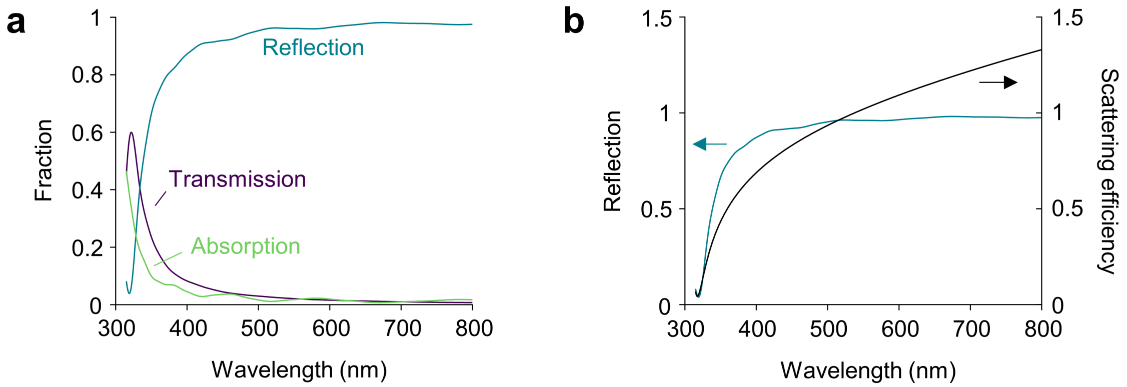

Figure S2: Analogy between the reflectivity of an Ag mirror and the scattering cross section of an Ag cylinder for longitudinal polarizations ($\vec{E}_{\parallel}$). (a) Transmission, reflection, and absorption of a 50 nm Ag film in air. (b) Comparison between the reflection of the 50 nm Ag film (black) and the scattering efficiency of an Ag cylinder with a radius of $R = 25$ nm illuminated with a polarization along its long axis (blue). The scattering efficiency is defined as $\sigma_{\text{sca}}/2R$.

3 Extinction of Ag cylinders with varying radii

The modelled extinction spectrum of an Ag cylinder with a radius $R = 25$ nm does not accurately reproduce the characteristic double peak that is experimentally observed (Figure 1 of the main text). Also for other values of R the extinction spectrum of an Ag cylinder does not show a double peak in the UV (Figure S3).

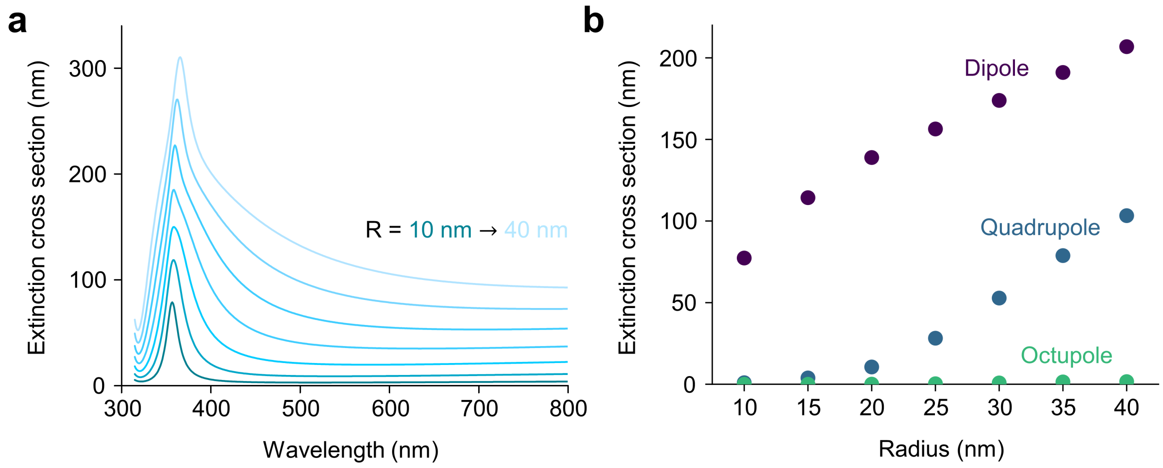

Figure S3: (a) Extinction spectra of Ag cylinders with different radii. The radius R is decreased from 10 nm (dark blue) to 40 nm (light blue) in steps of 5 nm. (b) Contributions from the dipolar (purple), quadrupolar (dark blue), and octupolar (light blue) modes to the extinction peaks in panel (a).

4 Full bandwidth comparison between circular and pentagonal cross sections

In Figure 1c,f of the main text we plot comparisons between simulated and measured extinction spectra for circular and pentagonal cross sections from 320 nm to 550 nm to highlight the transverse plasmon resonance. Below, in Figure S4, we plot the full simulated bandwidth (315 nm to 800 nm) to also highlight the residual extinction in the visible.

The extinction spectra are normalized to their maximum values and, therefore, the value of the normalized extinction in the visible is influenced by the height of the resonance peak in the ultraviolet. Small variations in the extinction in the visible can then be accounted for by changes in the resonance peak height, for example due to small variations in the radius of curvature (see Figure 1e in the main text) or in the imaginary part of the dielectric function.[2]

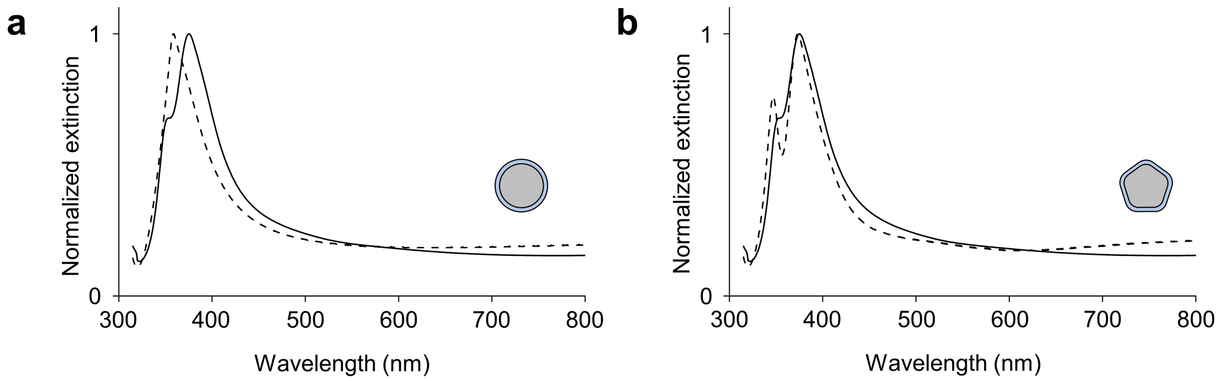

Figure S4: Comparison between the simulated (dashed) extinction spectrum of a circular (a) or pentagonal (b) infinite nanowire with the one measured experimentally for 25 nm radius PVP-stabilized AgNWs in water (solid).[3]

5 AgNW networks for solar cells

As can be seen in Figure 2b of the main text, the extinction peaks of AgNW networks lie outside the spectral range where the human eye is sensitive. Therefore, AgNW networks have a high transparency for transparent electrode applications (Figure S5a). However, solar cells also absorb in the UV, which coincides with the extinction peaks of the AgNW network. This overlap results in slightly lower transparency values when AgNW networks are used in solar cell applications (Figure S5b).

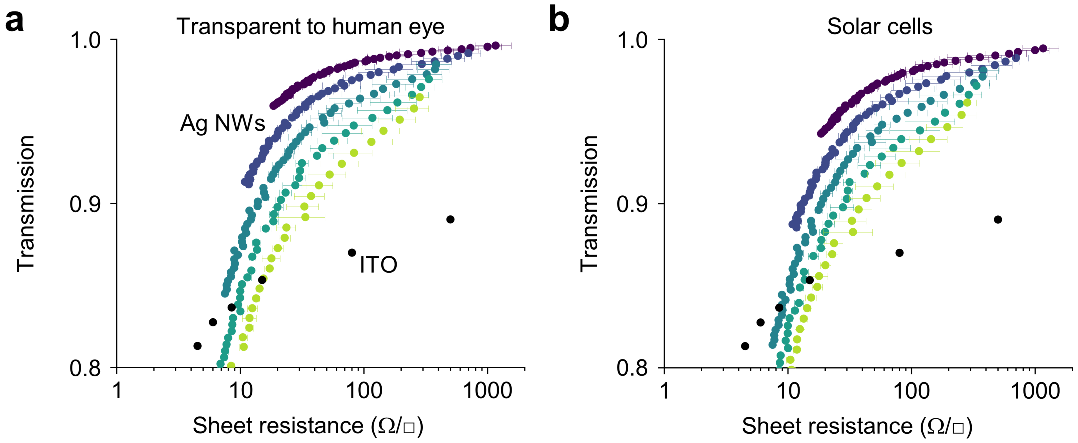

Figure S5: Transmission and sheet resistance values for AgNW networks for (a) transparent electrode and (b) solar cell applications. The source spectrum $I_0(\lambda)$ is the multiplication between the solar spectrum and the response of a typical human eye to light (a) or the portion of the solar spectrum with photon energies below the bandgap of Si, 1.12 eV (b). The sheet resistance simulation parameters are the same as those presented in Figure 2d of the main text.

6 AgNW network sheet resistance as a function of wire density

The model that we use for simulating the sheet resistance of an AgNW network takes into account both the resistance at the NW junctions and the resistance of the segments between the junctions.[4] When the junctions have poor electrical conductivity, the resistance in the NW segments does not contribute significantly to the sheet resistance. Therefore, in these cases, the sheet resistance is mostly dependent on the NW density, which determines the amount of junctions, and only weakly on the NW diameter, which determines the resistance of the segments (Figure S6a).

As the junctions become better and R_{junc} becomes lower, the sheet resistance of the network decreases and the influence of the NW segments becomes more dominant, which is demonstrated by the dependency of the sheet resistance on the wire radius R in Figure S6b,c.

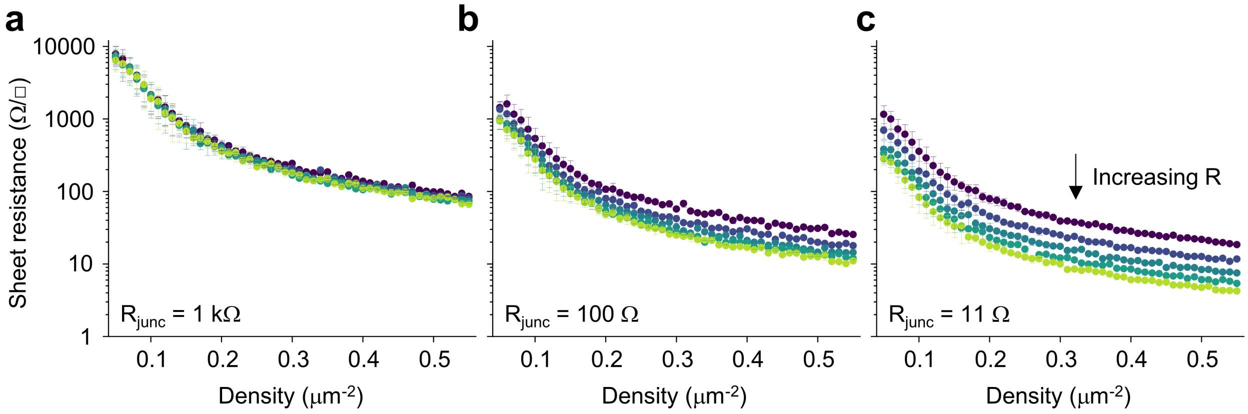

Figure S6: Simulated sheet resistance as a function of the AgNW density. The simulation parameters are the same as those presented in Figure 2d of the main text. The wire radius R is increased from 15 nm to 35 nm in steps of 5 nm. The junction resistance R_{junc} is 1 kΩ (a), 100 Ω (b), or 11 Ω (c).

7 Pentagonal vs circular cross section comparison

To calculate the area of a pentagon with rounded corners, we first define a triangle at one of the corners (Figure S7a). The area A_1 of this triangle is

$$A_1 = \frac{1}{2} R_{\text{curv}}^2 / \tan 54^\circ \tag{1}$$

The part of this triangle that is occupied with the rounded pentagon has an area

$$A_2 = \frac{36}{360} \pi R_{\text{curv}}^2 = \frac{\pi}{10} R_{\text{curv}}^2 \tag{2}$$

The amount of area that is taken away by rounding the corners then is

$$A_3 = 10(A_1 - A_2) \tag{3}$$

The initial unrounded pentagon has a radius R_{unr} that is larger than R (Figure S7a):

$$R_{\text{unr}} = R + R_{\text{curv}} \left(\frac{1}{\sin 54^\circ} - 1 \right) \tag{4}$$

The area of the rounded pentagon then becomes

$$A_4 = \frac{5}{4} \sqrt{\frac{5 + \sqrt{5}}{2}} R_{\text{unr}}^2 - A_3 \tag{5}$$

As can be seen in Figure S7b, when simulating cylinders and pentagons with equal cross-sectional areas, the resulting extinction spectra are similar for wavelengths above ∼450 nm. The characteristic double peak of the pentagon lies in the UV part of the spectrum, where solar irradiation is not intense and where the human eye is not sensitive (Figure 2b of the main text). Therefore, simulating pentagonal rather than cylindrical wires has no added benefit when calculating the transmission of AgNW networks for solar cell or transparent electrode applications (Figure 2c of the main text).

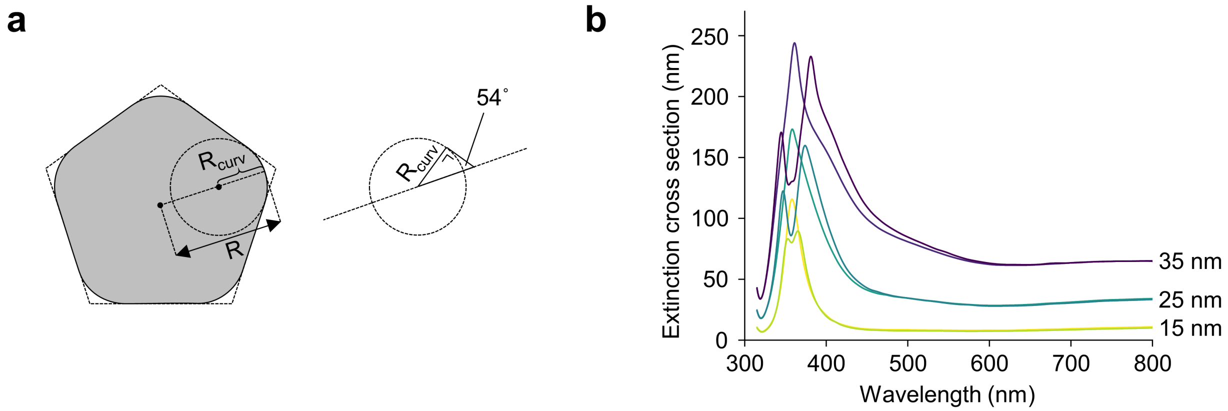

Figure S7: (a) Illustration indicating the radius R and radius of curvature R_{curv} of an AgNW with a pentagonal cross section. The triangle at one of the NW corners is used to calculate the cross-sectional area. (b) Extinction of wires with identical cross-sectional areas, but with a pentagonal ($R_{\mathrm{curv}} = 10$ nm) or circular cross section. The radii R are 15 nm, 25 nm, and 35 nm (pentagonal cross section), or 14.6 nm, 23.5 nm, and 32.3 nm (circular cross section).

References

(1) Jackson, J. D. *Classical Electrodynamics*; John Wiley & Sons, 2007.

(2) Yang, H. U.; D'Archangel, J.; Sundheimer, M. L.; Tucker, E.; Boreman, G. D.; Raschke, M. B. Optical Dielectric Function of Silver. *Phys. Rev. B* **2015**, *91*, 235137.

(3) Parente, M.; van Helvert, M.; Hamans, R. F.; Verbroekken, R.; Sinha, R.; Bieberle-Hutter, A.; Baldi, A. Simple and Fast High-Yield Synthesis of Silver Nanowires. *Nano Lett.* **2020**, *20*, 5759–5764.

(4) Manning, H. G.; da Rocha, C. G.; O'Callaghan, C.; Ferreira, M. S.; Boland, J. J. The Electro-Optical Performance of Silver Nanowire Networks. *Sci. Rep.* **2019**, *9*, 11550.
